# Efficacy of Tricaine Methanesulfonate (MS-222) as an Anesthetic Agent for Blocking Sensory-Motor Responses in *Xenopus laevis* Tadpoles

**DOI:** 10.1371/journal.pone.0101606

**Published:** 2014-07-01

**Authors:** Carlana Ramlochansingh, Francisco Branoner, Boris P. Chagnaud, Hans Straka

**Affiliations:** Department Biology II, Ludwig-Maximilians-University Munich, Planegg, Germany; University of Colorado, Boulder, United States of America

## Abstract

Anesthetics are drugs that reversibly relieve pain, decrease body movements and suppress neuronal activity. Most drugs only cover one of these effects; for instance, analgesics relieve pain but fail to block primary fiber responses to noxious stimuli. Alternately, paralytic drugs block synaptic transmission at neuromuscular junctions, thereby effectively paralyzing skeletal muscles. Thus, both analgesics and paralytics each accomplish one effect, but fail to singularly account for all three. Tricaine methanesulfonate (MS-222) is structurally similar to benzocaine, a typical anesthetic for anamniote vertebrates, but contains a sulfate moiety rendering this drug more hydrophilic. MS-222 is used as anesthetic in poikilothermic animals such as fish and amphibians. However, it is often argued that MS-222 is only a hypnotic drug and its ability to block neural activity has been questioned. This prompted us to evaluate the potency and dynamics of MS-222-induced effects on neuronal firing of sensory and motor nerves alongside a defined motor behavior in semi-intact *in vitro* preparations of *Xenopus laevis* tadpoles. Electrophysiological recordings of extraocular motor discharge and both spontaneous and evoked mechanosensory nerve activity were measured before, during and after administration of MS-222, then compared to benzocaine and a known paralytic, pancuronium. Both MS-222 and benzocaine, but not pancuronium caused a dose-dependent, reversible blockade of extraocular motor and sensory nerve activity. These results indicate that MS-222 as benzocaine blocks the activity of both sensory and motor nerves compatible with the mechanistic action of effective anesthetics, indicating that both caine-derivates are effective as single-drug anesthetics for surgical interventions in anamniotes.

## Introduction

Anesthetics have transformed modern medicine and basic biological research on vertebrate species by their ability to reversibly relieve pain, decrease body movements and render the animal unconscious during surgical interventions [1], [2]. While there are numerous potent, surgery-specific choices of anesthetics for use in mammals for which the mechanisms of action are well known [3], there is only a limited number of approved drugs for anesthesia in anamniote vertebrates such as fish and amphibians [4], [5], [6]. However, the increasing use of model species such as zebrafish, *Xenopus* or axolotl for systemic research approaches requires reliable and potent anesthetic drugs for which the sites and mechanisms of action are determined. Currently, the anesthetic of choice for fish and amphibian species is Tricaine methanesulfonate (MS-222), which is a sulfonated analog of benzocaine. Even though the pharmacological mechanism of benzocaine and its derivates are known on a cellular and subcellular level [7], [8], the precise mechanistic action and site(s)/extent of pharmacological impact on neuronal tissue on a systemic level *in vivo* remains relatively unclear and thus a distinction from related substances is elusive. This distinction, however, is of particular importance because a drug's classification strongly influences administration and usage policies in both medicine and basic research approaches that require approved animal protocols. Usually, during general surgical anesthesia in mammals a combination of several drugs cover analgesic, paralytic and anesthetic effects [9], while benzocaine and its derivates, as single substances, might cover all aspects during general anesthesia in fish and amphibians [5]. Assuming that caine-derivates potentially access all neuronal and myocytic elements during systemic applications, benzocaine or MS-222 could simultaneously block pain sensation [10], paralyze the animal by muscle relaxation and exert a general anesthesia [5] through a block of Na^+^-conductances [8] of cellular elements comprising the neuromuscular system (brain and muscles).

Benzocaine has been shown to effectively block the generation of action potentials through reversible interactions with voltage-dependent Na^+^-channels [1], [4], [5]. Consequently, this drug would be sufficient for anesthesia, analgesia and paralysis e.g. during surgical interventions in anamniote species, provided that the action potential initiation is blocked in all excitable cells, such as neurons and myocytes. Although MS-222 is a derivative and likely achieves its effect by a similar reversible suppression of action potentials, a complete understanding of the extent of this mechanism at the systemic level is still absent. As the current anesthetic of choice for fish and amphibians [5], MS-222 should block the action potential generation in both sensory and motor systems as well as in central nervous circuits. The sulfate moiety of tricaine (MS-222), which otherwise has a similar structure as benzocaine, renders the former drug much more hydrophilic [11]. The increased solubility has facilitated the employment as an anesthetic for poikilothermic vertebrates such as fish and amphibians [12], [13] because it allows administration simply by submerging these animals in an aqueous solution of the drug. This is at variance with benzocaine, which requires an organic solvent for its solubility [1]. After submergence in MS-222 (0.01–0.08%), a dose-dependent loss of the righting reflex and response to nociceptive stimuli was observed in leopard frogs [1] and decreased reactions to noxious stimuli have been reported in koi fish [10]. The impact on motor activity as well as on nociception suggests that along with the anesthetic effect, MS-222 as a single drug is well suited for surgical interventions in anamniote vertebrates. However, the potency of this drug on the spike discharge of distinct neuronal populations or on the activity of identified neural networks responsible for the mediation of a defined sensory-motor behavior still remains to be determined.

Here, we describe the dose-dependent effect of MS-222 on spontaneous and evoked activity in afferent sensory (vestibular, lateral line) as well as in motor (extraocular) nerves in *Xenopus laevis* tadpoles. In semi-intact *in vitro* preparations with functional sensory organs and motor effectors [14] the anesthetic impact and pharmacological capacity of MS-222 was determined at the input and output stage of a well defined sensory-motor behavior in comparison to benzocaine and pancuronium (a pure paralytic).

## Material and Methods

### Animals

*Xenopus laevis* tadpoles were obtained from the in house animal breeding facility at the Biocenter-Martinsried (Ludwig-Maximilians-University Munich). Tadpoles were kept in tanks filled with 17–18°C non-chlorinated water at a 12/12 light/dark cycle and were fed daily with finely ground algae. A total of 89 animals at developmental stages 53–57 [15] were used for experimentation. Experiments were performed *in vitro* on isolated, semi-intact preparations and comply with the National Institute of Health publication entitled "Principles of animal care", No. 86–23, revised 1985. Permission for these experiments was granted by the governmental institution, Regierung von Oberbayern/Government of Upper Bavaria (55.2-1-54-2532.3-59-12).

### Isolated semi-intact in vitro preparation

**Figure 1:**
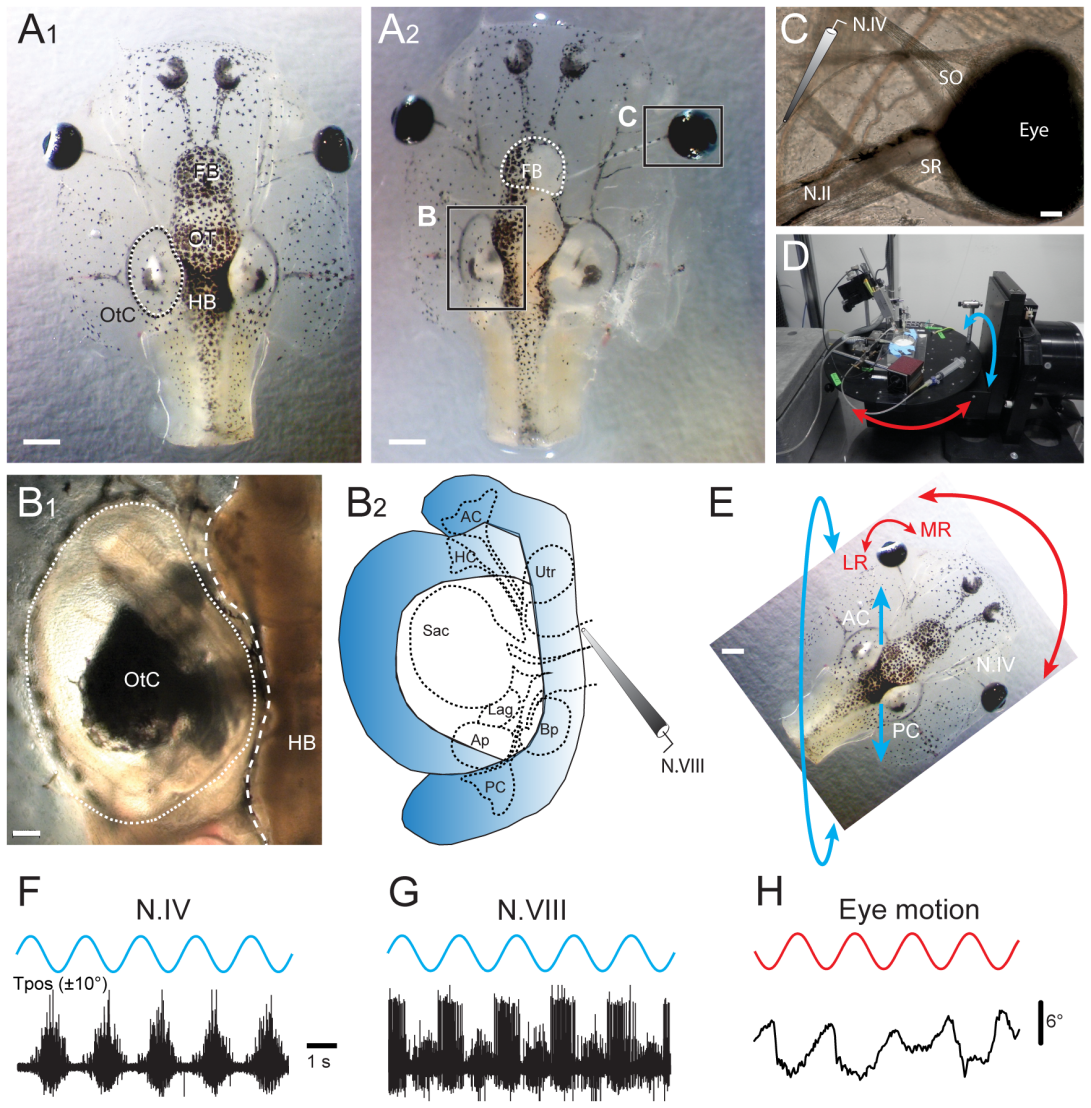
Semi-intact *Xenopus laevis* preparation with functional sensory and motor elements for *in vitro* pharmacological experimentation. ***A-C***, Photomicrographs, depicting an isolated head of a *Xenopus laevis* tadpole before (***A_1_***) and after opening of the dorsal cranium (***A_2_***) and removal of the skin to disconnect the forebrain (FB, white dotted line in ***A_2_***) and to make the brainstem, otic capsule (OtC in **A_1_,** ***B_1,2_***) and extraocular neuromuscular system (i.e. N.IV motor innervation of the SO eye muscle in ***C***) accessible for extracellular single- and multiple-unit recordings and bath-application of drugs; further removal of the entire hindbrain (HB, white dashed line in ***B_1_***) yielded access to the peripheral part of the N.VIII for electrophysiological recordings of afferent fibers innervating the different vestibular sensory endorgans in the otic capsule (***B_2_***). ***D,E***, A computer-controlled turntable (***D***) allowed spatially specific application of sinusoidal rotational stimuli of semi-intact preparations in the horizontal (red arrows) and vertical planes (e.g. left AC - right PC, blue arrows in ***E***). ***F,G***, Typical multiple-unit discharge modulation, recorded from N.IV (trochlear nerve, ***F***) and the anterior branch of N.VIII (vestibular nerve, ***G***) during 5 cycles of rotation (0.5 Hz) in the AC - PC plane, illustrated in ***E***. ***H***, *In vitro* optical motion recording of the left eye (***E***) during 5 cycles of rotation in the horizontal plane (0.5 Hz). Scale bar is 1 mm in ***A_1,2_*** and 0.2 mm in ***B_1_,C***; calibration bar and positional excursion amplitude of the rotational stimulus in ***F*** also apply to ***G,H***; AC, PC, anterior, posterior vertical semicircular canal; LR, MR, SR, lateral, medial, superior rectus eye muscle; SO, superior oblique eye muscle; OT, optic tectum, Tpos, table position.

In all experiments, tadpoles were anesthetized in 0.05% MS-222 (Pharmaq Ltd., UK) in frog Ringer (75 mM NaCl, 25 mM NaHCO_3_, 2 mM CaCl_2_, 2 mM KCl, 0.5 mM MgCl_2_, and 11 mM glucose, pH 7.4) and decapitated at the level of the upper spinal cord. For experiments on the lateral line system, the spinal cord was cut at the same segmental level, however, the entire tail musculature and skin remained intact. As previously described [16], the skin covering the dorsal head (Fig. 1A_1_) was removed, the soft skull tissue opened and the forebrain disconnected (dotted white line in Fig. 1A_2_). This surgical procedure anatomically preserved the bilateral vestibular endorgans within the otic capsule (inner ear; Fig. 1B_1,2_), the central nervous system (brain) and the extraocular motor innervation and eye muscles (Fig. 1C) for prolonged *in vitro* experimentation [14]. The functionally persisting central nervous system, intact vestibular sensory periphery, afferent connections and efferent projections to the eye muscles along with spatio-temporally appropriate contractions of the latter allowed an *in vivo*-like activation of vestibulo-ocular reflexes (VOR) during natural motion patterns (Fig. 1D,E) under controlled *in vitro* conditions [17], [14]. This isolated preparation is thus highly suitable for pharmacological studies because it combines the advantages of *in vitro* experiments for bath application of defined drug concentrations with the possibility to quantitatively determine the impact of a particular drug on sensory and motor behavior, otherwise only possible under *in vivo* conditions.

### Neuronal activity and behavioral measurements

Spontaneous and evoked neural activity was recorded from selected sensory and motor nerves along with motion-triggered eye movements (Fig. 1F-H) [16]. After the isolation and completion of the final dissection that gave access to individual target nerves (vestibular, posterior lateral line, trochlear) all preparations were rinsed in freshly oxygenated Ringer solution (Carbogen: 95% O_2_, 5% CO_2_), transferred to a small tank (volume 40–50 ml) and maintained for 24 hours at 6–8°C to ensure complete wash-out of MS-222 after the different preparations had been generated (description, see below).

*Vestibular nerve (N.VIII)*

Both N.VIII were transected at the entrance into the brainstem, leaving the peripheral part of each nerve along with the ganglion of Scarpa and the sensory innervation of individual vestibular endorgans in the otic capsule intact (Fig. 1B_1,2_). The brain was removed to facilitate the visibility and access to the cut surface of the N.VIII for electrophysiological recordings. The next day, the preparation was mechanically secured to the Sylgard floor of a recording chamber that was mounted onto a two-axis turntable (Fig. 1D) in the center of the horizontal and vertical rotation axes. The spike discharge of vestibular nerve afferent fibers was recorded by targeting glass suction electrodes with a micromanipulator to the central portion of the fasciculated cut end of the N.VIII (Fig. 1G).

#### Lateral line nerve

The posterior lateral line nerve was severed at the entrance into the brain. This made the peripheral portion of the nerve, connected to the sensory periphery of the lateral line system, accessible for electrophysiological recordings. A large part of the peripheral sensory target area of the posterior lateral line nerve (neuromasts on the skin of the dorsolateral part of the head and rostral tail) remained intact and allowed natural stimulation through induction of water waves along the body surface. Electrophysiological recordings were made on the following day from the posterior lateral line nerve with individually adjusted glass suction electrodes.

#### Trochlear nerve (N.IV)

The skin surrounding the eyes was removed and the identified nerve branch innervating the superior oblique (SO) eye muscle, i.e. the trochlear nerve (Fig. 1C) was disconnected from its target muscle. After 24 hours the preparation was fixed to the Sylgard floor in the recording chamber, which was mounted in the center of the rotation axes of the turntable as described above (Fig. 1D,E). Individually adjusted glass suction electrodes were used for the electrophysiological recordings as in earlier studies (Fig. 1F) [18], [16].

#### Electrophysiological recordings of nerve activity

Spontaneous and sensory stimulus-evoked multiple-unit spike discharge of the vestibular, lateral line and trochlear nerve before, during and after drug application were recorded extracellularly (EXT 10-2F; npi electronics, Germany) with glass suction electrodes, digitized at 10–20 kHz (CED 1401, Cambridge Electronic Design, UK) and stored on computer for offline analysis. Glass microelectrodes for extracellular recordings were made with a horizontal puller (P-87, Sutter Instruments Co., USA) and were individually broken at the tip to fit the respective nerve diameters.

#### Eye motion

After isolation of the preparation and maintenance in oxygenated Ringer solution for 24 hours, the recording chamber with the preparation secured to the Sylgard floor was mounted on the two-axis turntable in the center of the rotation axes (Fig. 1D,E). Sinusoidal rotations around the vertical axis triggered oppositely directed compensatory eye movements in the horizontal plane, illustrating the activation of an angular VOR [19]. Even though this *in vitro* preparation is isolated, it is still capable of expressing effective motor reactions upon sensory stimulation, such as compensatory eye movements during vestibular stimulation on motion simulators with spatio-temporal characteristics identical to those observed in intact animals [14], [18]. Eye movements were recorded non-invasively (Fig. 1H) with a video camera (Grasshopper B/W, Point Grey Research Inc, Canada) and a zoom objective (Optem Zoom 70XL, Qioptiq Photonics GmbH & Co KG, Germany) with an adequate lens (M25×0.75+0.25). This system was mounted on top of the experimental setup to visualize the motion of one or both eyes from above during vestibular stimulation at a video capture frame rate of 30 Hz with the respective software (FlyCap2 v2.3.2.14.).

During all electrophysiological and eye motion recordings, preparations were continuously superfused with oxygenated Ringer solution at a rate of ∼2 ml/min. The temperature of the bath solution was controlled and maintained at 17±0.5°C throughout the experiments.

### Sensory stimulation

Motion stimuli for modulating vestibular (N.VIII) nerve afferent and trochlear (N.IV) motor nerve activity were delivered with a computer-controlled motorized two-axis turntable (Fig. 1D; Acutronic Switzerland Ltd.), suitable to cause natural motion-dependent activation of the vestibular endorgans. The stimulus consisted of sinusoidal rotations around the horizontal axis at a frequency of 0.5 Hz and position amplitude of ±10° (blue arrows in Fig. 1E) corresponding to a peak table velocity of ±30°/s. The recording chamber with the isolated preparation in the center was positioned on the turntable such that the plane of the turntable rotation (roll stimulation) caused a maximal discharge modulation of the vestibular afferent activity in a particular experiment. During trochlear motor nerve recordings, the roll motion stimulation occurred along the ipsilateral posterior/contralateral anterior semicircular canal plane to maximize spike discharge modulation of these extraocular motoneurons [19]. For eliciting horizontal VOR-associated eye movement behavior, the motion stimulus consisted of a vertical-axis rotation at a frequency of 0.5 Hz and a positional excursion of ±10° corresponding to a peak table velocity of ±30°/s and specifically activated the bilateral horizontal semicircular canals (red arrows in Fig. 1E).

Water waves, produced with a vibrating sphere (diameter 3 mm) were used to activate lateral line hair cells of neuromasts on the skin surface. The sphere was positioned with a micromanipulator lateral to the tail of the animal and controlled by a piezo drive (PX 1500, Piezosystem Jena, Germany). The vibrating sphere was cyclically displaced along the rostro-caudal body axis at the level of the tail at a distance of ∼1 mm from the skin surface at a frequency of 10 Hz, a displacement of ±150 µm for a period of 10 s. Spike discharge activity in the posterior lateral line nerve, innervating the respective neuromasts, was measured for a period of 10 s prior and during stimulation.

### Pharmacological agents

The drugs, MS-222 (Pharmaq Ltd., UK), benzocaine and pancuronium (both from Sigma-Aldrich, France), were administered to the preparation using bath application of the respective substance in oxygenated Ringer solution at a rate of ∼2 ml/min, at a temperature of 17°C [17]. MS-222 at four different concentrations (0.0025, 0.005, 0.01 and 0.05%) and pancuronium (0.12 mg/ml) were dissolved in frog Ringer. In contrast, benzocaine was dissolved in 0.003% dimethylsulfoxide (DMSO, Sigma-Aldrich, France) before dilution in frog Ringer (final concentrations 0.0025, 0.005, 0.01 and 0.05%). All drug solutions were buffered to a pH 7.4 with NaHCO_3_. A drug-related reduction of the spontaneous and evoked responses in similar isolated preparations usually occurred after 3–5 min and reached steady state after 15 min [20]. The wash-out of the different drugs was determined for a period of up to four hours in some experiments and revealed a dose- and drug-dependent recovery of the resting rate and stimulus-induced discharge modulation.

### Analysis

Eye motion profiles and parameters were extracted from the captured video sequences using a custom video-processing algorithm written in Matlab [21]. To calculate the motion of the eye, an ellipse was drawn around the eyeball and the angle between the minor axis of the ellipse and the longitudinal axis of the head was calculated in each frame of a given video sequence. Based on the frame rate (30 Hz), the change in eye position over time was computed. The generation of peri-stimulus time histograms for a single cycle of sinusoidal vertical-axis turntable rotation was used to calculate the eye motion gain (ratio eye/table motion) of the VOR. This value was determined before (control), during and after drug application (wash-out) to measure the impact of the different substances. Electrophysiological recordings were analyzed with Spike2 software using customized scripts to create peri-stimulus time histograms (PSTH). The PSTH values were grouped into control, 5 and 15 min exposure and wash-out after drug removal. All values were for comparative reasons normalized to control values obtained prior to the drug application. The normalized values were statistically analyzed using one-way ANOVA and post-hoc tests (Dunnett and Tukey's; GraphPad Prism software) to assess significance levels. Averaged results were expressed as means ± standard error of the mean (SEM). Graphical presentations were made with the aid of commercially available computer software (GraphPad Prism; Igor Pro; Illustrator, Adobe Systems).

## Results

### Effect of MS-222, benzocaine and pancuronium on eye motion

**Figure 2:**
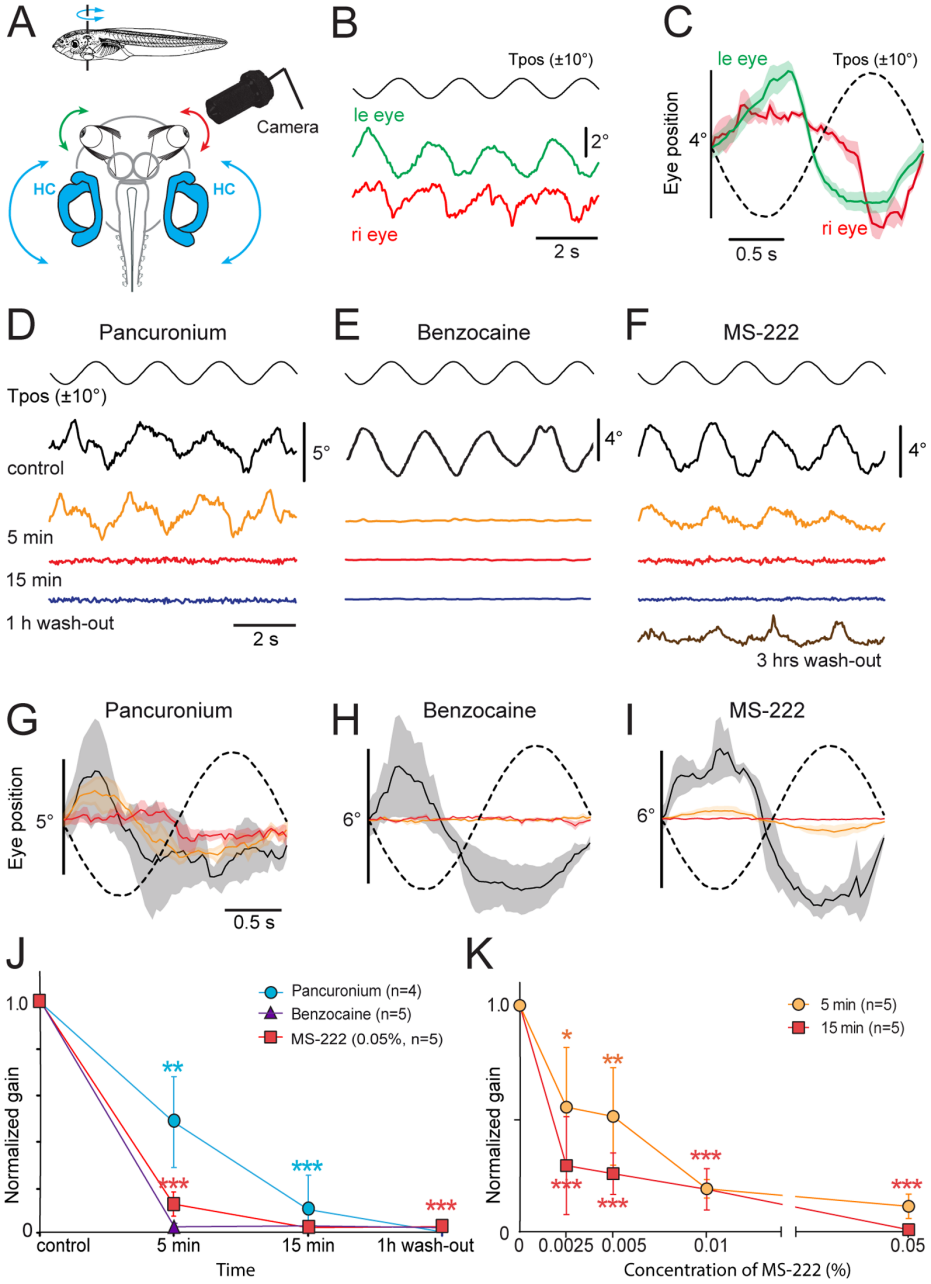
Effect of pancuronium, benzocaine and MS-222 on the horizontal aVOR in *Xenopus* tadpoles. ***A***, Schematic of the experimental setting depicting the semi-intact preparation, horizontal sinusoidal turntable positional changes (Tpos; 0.5 Hz, ±10°) and video capture of eye motion. ***B,C***, Single sweeps of co-aligned horizontal positional oscillations of the left (green) and right (red) eye, extracted from video sequences during turntable rotation (***B***) and average responses (± SEM, shaded areas; *n* = 8) over a single cycle (***C***); note that table and eye motion are oppositely directed in agreement with aVOR behavior. ***D-I***, Effect of bath-applied pancuronium (0.12 mg/ml, ***D,G***), benzocaine (0.05%, ***E,H***) and MS-222 (0.05%, ***F,I***) on single sweeps of horizontal sinusoidal turntable motion (Tpos)-evoked eye movements (***D-F***) and average response (± SEM, shaded areas; *n* = 8, respectively) over a single cycle (dashed line; ***G-I***); recordings (same color code in ***G-I***) were obtained before (Control, black traces), 5 min (orange traces) and 15 min (red traces) after onset of drug application as well as 1 hour (blue traces, ***D-F***) and 3 hours after beginning of the wash-out (brown trace, ***F***). ***J***, Progression of pharmacological influence on eye motion gain (ratio of eye/table motion magnitude) after application/wash-out of pancuronium, benzocaine and MS-222. ***K***, Dose-response curve of MS-222 impact on the gain of rotation-induced eye motion 5 and 15 min after drug application; the significance of difference in ***J,K*** is indicated: *, *p*≤0.01; **, *p*≤0.001; ***, *p*≤0.0001, with respect to control values. Calibration bars in ***D,G*** also apply to ***E,F*** and ***H,I***, respectively.

Application of sinusoidal rotational stimuli to isolated semi-intact *in vitro* preparations of *Xenopus* tadpoles with a motion simulator (Figs. 1A,D,E, 2A) activates a robust vestibulo-ocular reflex behavior with characteristics comparable to those observed *in vivo* [14], [18]. Accordingly, non-invasive video recordings of compensatory eye movements during sinusoidal rotation (0.5 Hz; ±10°) around the vertical axis in control animals revealed conjugate movements of both eyes in the opposite direction to the turntable motion (Figs. 1H, 2B,C) with a gain (ratio eye/table motion) of 0.25±0.04 (*n* = 20), typical for the horizontal angular VOR (aVOR) in larval *Xenopus* at this developmental stage [18].

Following bath application of pancuronium (0.12 mg/ml), a paralytic drug [22], the evoked eye movements were gradually reduced, reached ∼50% of the control values after 5 min and were essentially abolished (*n* = 4) after 15 min (Fig. 2D,G,J). The block of the response was long-lasting as indicated by the absence of any recovery of the responses 1 hour after the beginning of the wash-out of the drug (Fig. 2D,G,J). As expected from the pharmacological mechanism of pancuronium as a competitive nicotinic acetylcholine receptor antagonist [22], vestibular-evoked extraocular muscle activity was consistently blocked for an extended period in isolated *Xenopus* tadpole preparations.

Bath application of benzocaine (0.05%), a reversible blocker of voltage-dependent Na^+^ channels [8] exhibited a very potent and rapid effect on the evoked aVOR behavior (Fig. 2E,H,J). Motion-triggered eye movements during sinusoidal turntable rotation were essentially blocked 5 min after the initial application in all preparations (*n* = 5) and were consistently extinguished 15 min after adding the drug to the bath solution (Fig. 2E,H,J). At a concentration of 0.05%, no recovery was seen 1 hour after wash-out (Fig. 2E,H,J) and the block usually persisted for several hours. The faster onset of the effect, relative to pancuronium, is likely due to the ubiquitous accessibility of Na^+^ channels in all neurons along the VOR pathway in this preparation, thereby blocking the activity at the sensory periphery in the inner ear, the central vestibular and extraocular motor nuclei as well as the eye muscles, in agreement with the expected pharmacological impact of this drug.

Bath application of MS-222 at a concentration of 0.05% exhibited a slightly slower time course and lower potency of blocking the elicited aVOR behavior compared to benzocaine (Fig. 2E,F,H-J). Nonetheless, five minutes after bath application of MS-222 (0.05%), vestibular-evoked eye movements were reduced by ∼90% (*n* = 5) and completely blocked 15 min after adding the drug to the bath solution (Fig. 2F,I,J). No detectable recovery of the aVOR behavior was seen after 1 hour wash-out at this concentration, however, a partial recovery was observed 3 hours after removal of MS-222 from the bath (Fig. 2F,I,J). The effect of this drug on the evoked aVOR behavior was dose-dependent as indicated by the more pronounced effects of increasing concentrations on the response, both 5 and 15 min after wash-in of the drug (Fig. 2K). While a significant reduction of the eye motion amplitude was caused at all concentrations, a complete block of the responses 15 min after application was only achieved at a concentration of 0.05% MS-222 (Fig. 2K).

### Effect of MS-222, benzocaine and pancuronium on spontaneous and evoked extraocular motor activity

**Figure 3:**
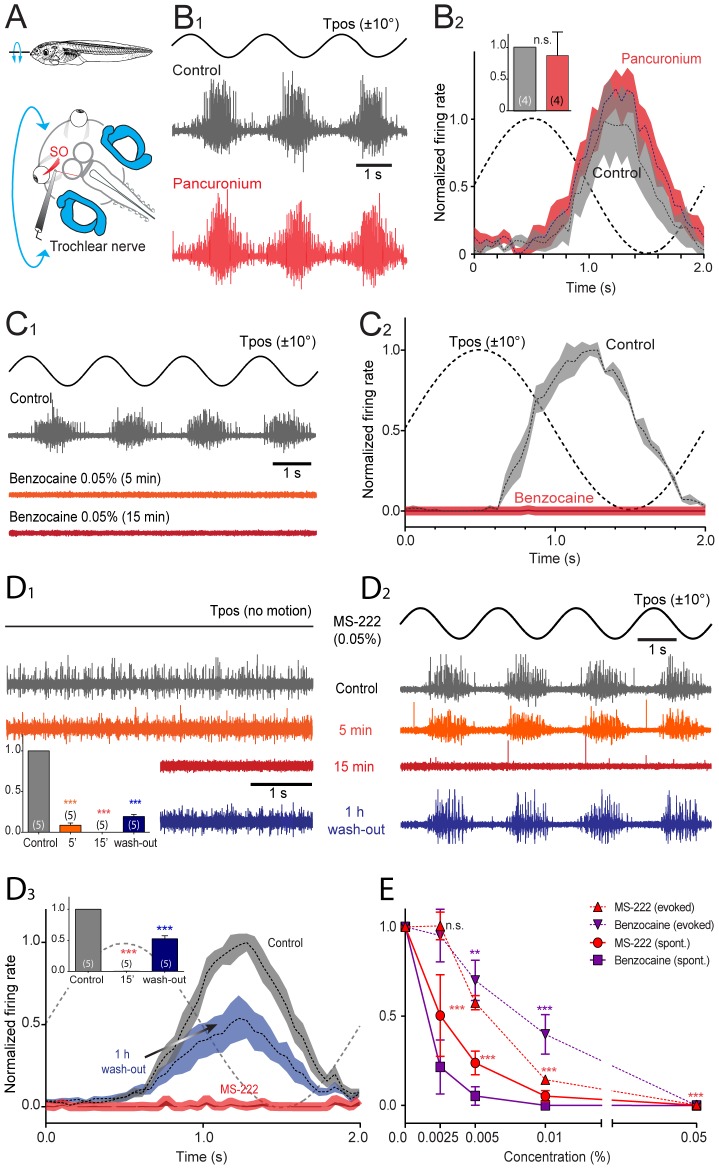
Effect of pancuronium, benzocaine and MS-222 on extraocular motoneuronal discharge in *Xenopus* tadpoles. ***A***, Schematic of the experimental setting depicting the semi-intact preparation, roll-axis turntable positional changes (T_pos_, 0.5 Hz, ±10°) and extracellular recordings of spontaneous and motion-evoked multiple-unit discharge of the superior oblique (SO) eye muscle-innervating trochlear nerve. ***B-D***, Single sweeps of sinusoidal roll motion (Tpos)-evoked discharge modulation (***B_1_***,***C_1_***,***D_2_***), spontaneous firing of the trochlear nerve (***D_1_***) and average firing rate modulation (± SEM, shaded areas) over a single cycle (dashed line; ***B_2_***,***C_2_***,***D_3_***) before (Control, gray traces and plots), 5 min (orange traces and plots), 15 min (red traces and plots) and after wash-out (blue traces and plots) of bath-applied pancuronium (0.12 mg/ml, ***B***), benzocaine (0.05%, ***C***) and MS-222 (0.05%, ***D***); inset in ***B_2_*** shows relative peak firing rates before (gray) and after pancuronium application (red); insets in ***D_1,3_*** show relative resting rates (***D_1_***) and peak firing rates during roll motion stimulation (***D_3_***) before and during drug application and after wash-out; number of experiments in parentheses in insets; while no significant change (*n.s.*, *p* = 0.975) of the peak firing rate occurred after adding pancuronium to the bath (***B***), benzocaine (***C***) and MS-222 (***D***) blocked the motor responses entirely (***, *p*≤0.0001). ***E***, Dose-response curve of benzocaine and MS-222 (*n* = 20, respectively) on spontaneous and motion-triggered peak modulation values of multiple-unit trochlear motor spike discharge.

In order to comparatively evaluate the effect of the different paralytic and anesthetic drugs on motor commands that generate aVOR behavior, the neuronal discharge of the respective motoneurons was recorded from the trochlear nerve during natural vestibular stimulation (Fig. 3A). After disconnecting the trochlear nerve from its target superior oblique extraocular muscle, extracellular multiple-unit spike activity was recorded at rest and during horizontal-axis roll stimulation in the plane of the ipsilateral posterior/contralateral anterior vertical semicircular canal (Fig. 3A,B_1_). A rotation along this plane optimally activates this eye muscle [18]. In the absence of table rotation the rate of the multiple-unit trochlear nerve (N.IV) activity was ∼15 Hz (14.6±2.6 Hz; *n* = 14). During sinusoidal turntable motion (0.5 Hz; ±10°) in the roll plane, the extraocular motor nerve discharge was modulated with a dynamic characteristic that matched the expected motor command signal for compensatory eye movements in isolated preparations with intact neuro-muscular connections [18]. The average peak discharge modulation rate during roll motion stimulation was ∼140 Hz (140.6±21.4 Hz; *n* = 14), with the peak firing rate occurring slightly before the table position tilted in the direction of the ipsilateral posterior semicircular canal (Fig. 3B_1,2_,C_1,2_).

Bath application of pancuronium (0.12 mg/ml; *n* = 4), had neither a significant effect on the spontaneous trochlear nerve firing rate (*p* = 0.918) nor on the discharge modulation during turntable motion (*p* = 0.975) compared to controls (Fig. 3B_1,2_). The insensitivity of the extraocular motor nerve discharge to pancuronium (Fig. 3B_2_), at variance with the suppression of eye movements by this drug during the same stimulus (Fig. 2D,G,J) complies with the absence of nicotinic acetylcholine receptors in central VOR pathways [19]. This shows that the observed block of aVOR behavior (Fig. 2D,G) was caused by the impact of pancuronium on the synaptic transmission at the neuromuscular synapse between extraocular motoneurons and eye muscles.

**Figure 4:**
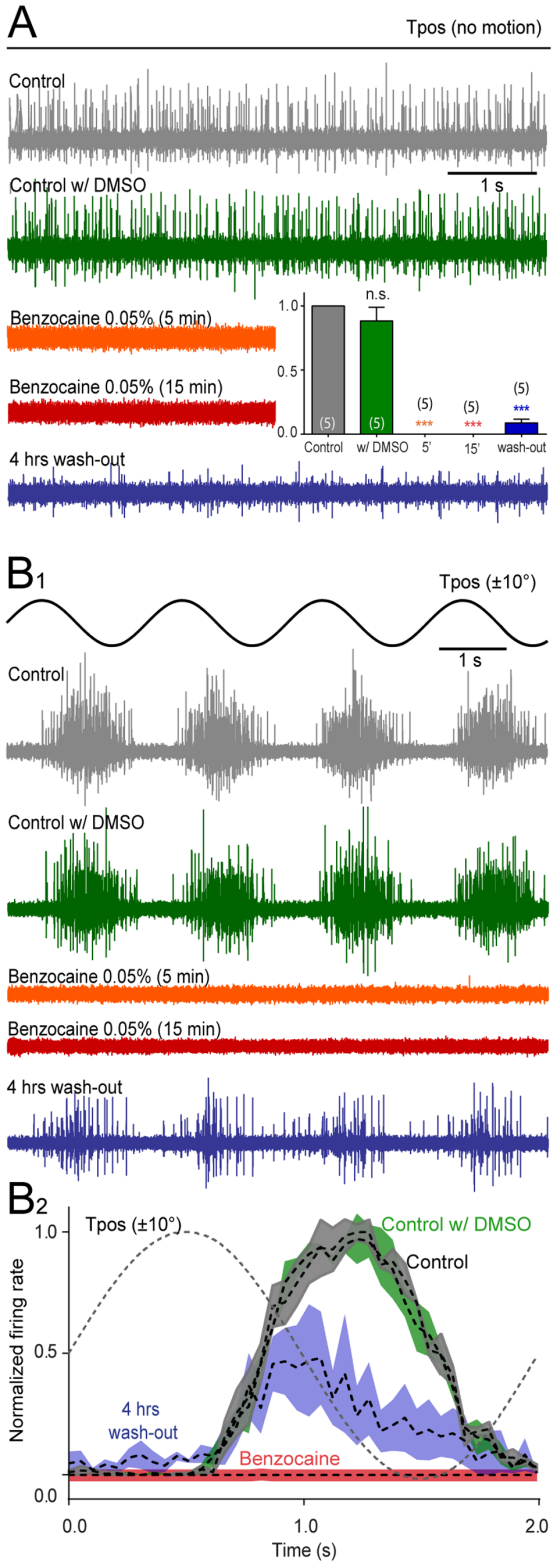
Effect of benzocaine on extraocular motoneuronal discharge in *Xenopus* tadpoles. ***A-B***, Single sweeps of spontaneous (***A***), roll motion (Tpos)-evoked (***B_1_***) multiple-unit discharge of the superior oblique (SO) eye muscle-innervating trochlear nerve and average firing rate modulation (± SEM, shaded areas; ***B_2_***) over a single cycle (dashed line; ***B_2_***) before (Control, gray traces and plot), 10 min after application of dimethylsulfoxide (Control w/DMSO, green traces and plot), 5 min (orange traces) and 15 min after application of benzocaine (0.05%; red traces) and four hours after wash-out (blue traces and plot); inset in ***A*** show relative resting rates before (gray), in the presence of DMSO (green), benzocaine (orange and red, complete block) and after wash-out (blue); number of experiments in parentheses; while no significant change (*n.s.*, *p* = 0.880) of the peak firing rate occurred after adding DMSO to the bath, benzocaine (0.05%) blocked the motor responses entirely (***, *p*≤0.0001).

Bath application of benzocaine, in contrast to pancuronium, blocked the spontaneous multiple-unit discharge as well as the modulated activity of the trochlear nerve during roll motion stimulation (Figs. 3C_1,2_, 4A,B). Following application of benzocaine (0.05%; *n* = 5) to the bath solution, the spontaneous multiple-unit firing (Fig. 4A) as well as the discharge modulation during sinusoidal roll motion (Figs. 3C_1,2_, 4B_1,2_) ceased rapidly and caused a complete and long-lasting block of the spike activity in the nerve in all experiments after 5 min (Figs. 3C_1,2_,E, 4B_1,2_). During wash-out, both the spontaneous activity as well as the vestibular-evoked modulation of the trochlear nerve discharge remained suppressed for more than 1 hour and only reappeared gradually thereafter (Fig. 4A,B).

Bath application of MS-222 (0.05%; *n* = 5) was similarly effective in blocking the trochlear nerve activity (Fig. 3D) as benzocaine. After adding MS-222 to the bath solution, the spontaneous multiple-unit firing rate gradually ceased, reached ∼10% of the control value after 5 min and was completely blocked after 15 min for a prolonged period (Fig. 3D_1_,E). In contrast to the relatively rapid blocking effect of this drug on the resting rate in the absence of motion, the peak discharge during roll motion stimulation diminished more slowly and reached ∼60% of the control value after 5 min but was completely blocked after 15 min in all experiments (Fig. 3D_2,3_,E). During wash-out of MS-222, the spontaneous discharge as well as the motion-evoked firing rate modulation depth recovered slowly and reached 20 and 50%, respectively, of the initial magnitudes after 1 hour (insets in Fig. 3D_1,3_) and was generally re-established after 4 hours.

In order to comparatively assess the potency and dynamics of the benzocaine and MS-222-induced reduction of the spontaneous and evoked trochlear nerve discharge, the suppressive influence on motor activity was tested after bath-application of the two drugs at four different concentrations (Fig. 3E). Since benzocaine was dissolved in DMSO, a control application of this solvent in the absence of benzocaine was performed and found to have no influence on the spontaneous and motion-evoked trochlear nerve firing rate (*p* = 0.880; Fig. 4A,B). Comparison of the dose-dependency indicated that benzocaine is more potent than MS-222 with regard to spontaneous activity since application of the former drug achieved a complete block of the nerve discharge at lower concentrations compared to the latter (Fig. 3E). However, both drugs effectively and reliably suppressed the spontaneous and evoked extraocular motor activity in a similar time-dependent and reversible manner, consistent with the expected mechanistic action of this class of anesthetic drugs.

### Effect of MS-222 on spontaneous and evoked sensory nerve activity

The clear, dose-dependent and reversible suppressive effect of MS-222 on spontaneous and sensory-evoked extraocular motor responses is likely due to drug actions at multiple levels along the VOR pathway. However, in order to evaluate and isolate the potential suppressive effect of this drugs on the discharge of sensory fibers, a series of experiments was conducted that determined the potency and dynamics of this drug on mechanoreceptor-related (vestibular, lateral line) afferent sensory activity.

#### Lateral line nerve activity

**Figure 5:**
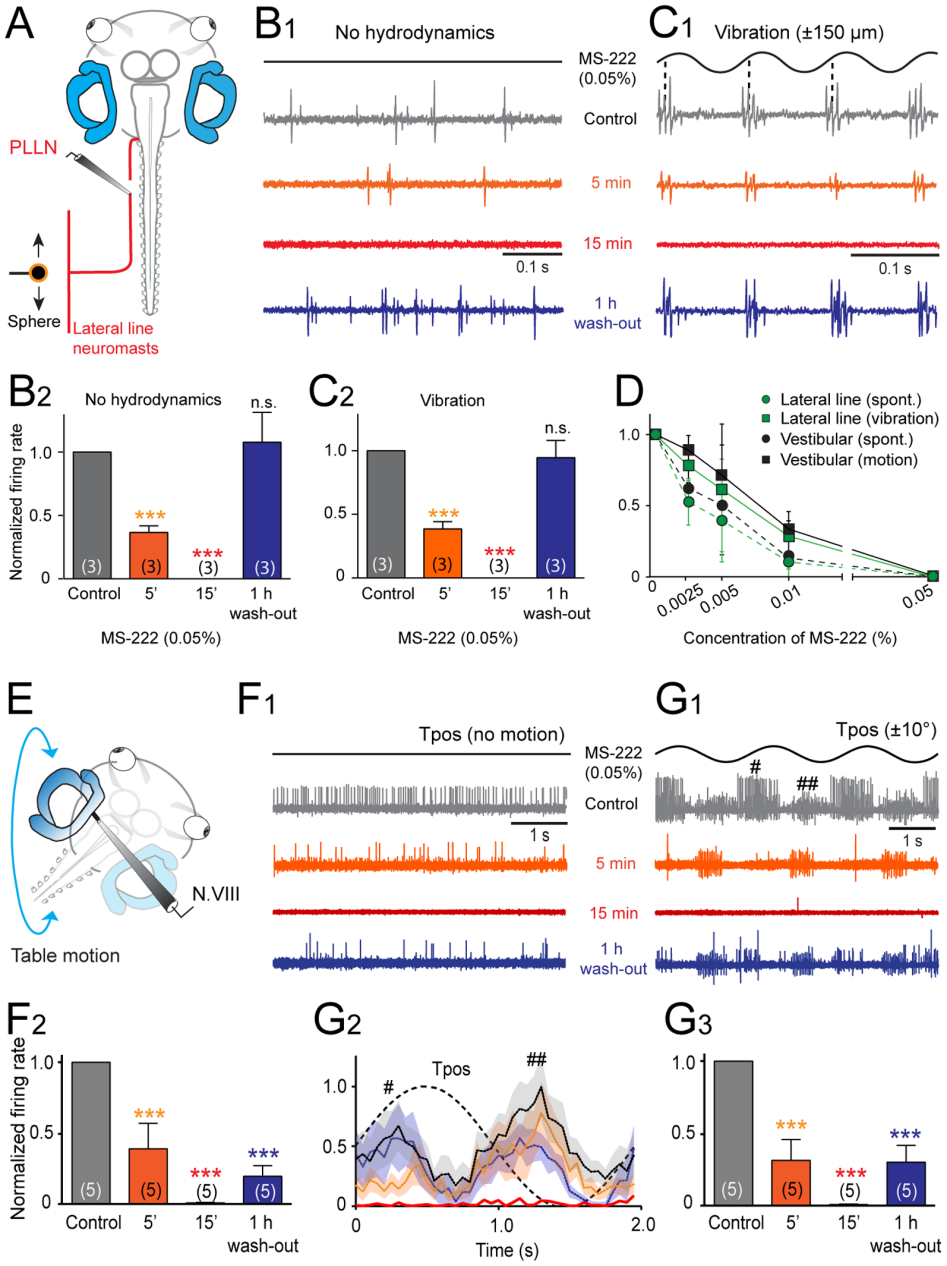
Effect of MS-222 on the spike discharge of mechanoreceptor sensory afferent fibers in *Xenopus* tadpoles. ***A***, Schematic of the experimental setting depicting the semi-intact preparation, vibrational stimulation of lateral line neuromasts (10 Hz, ±150 µm) and extracellular recordings of the multiple-unit discharge of the peripheral portion of the posterior lateral line nerve (PLLN). ***B,C***, Single sweeps of spontaneous (***B_1_***) and vibration-evoked discharge modulation (***C_1_***) in the PLLN before (Control, gray traces and plots), 5 min (orange traces and plots) and 15 min after bath-application (red traces and plots) and after wash-out (blue traces and plots) of MS-222 (0.05%); relative resting (***B_2_***) and peak firing rates (***C_2_***) before, during and after wash-out show the reversible drug effect; number of experiments in parentheses. ***D***, Dose-response curve of MS-222 on multiple-unit spontaneous and sensory stimulus-evoked spike discharge modulation in mechanosensory (lateral line and vestibular) nerve fibers. ***E***, Schematic of the experimental setting depicting the semi-intact preparation, roll-axis turntable positional changes (T_pos_, 0.5 Hz, ±10°) and extracellular recordings of the multiple-unit discharge of the peripheral portion of N.VIII (vestibular). ***F,G***, Single sweeps of spontaneous (***F_1_***) and roll motion-evoked discharge modulation (***G_1_***) of the N.VIII and average firing rate modulation (± SEM, shaded areas; *n* = 8, respectively) over a single cycle (dashed line; ***G_2_***) before (Control, gray traces and plots), 5 min (orange traces and plots) and 15 min after bath-application (red traces and plots) and after wash-out (blue traces and plots) of MS-222 (0.05%); ^#^,^##^ in ***G_1,2_*** indicate activated responses in different nerve fibers by either direction of the sinusoidal roll motion; relative resting (***F_2_***) and peak firing rates (***G_3_***) before, during and after wash-out show a complete block and partially reversed drug effect; number of experiments in parentheses; ***, *p*≤0.0001; *n.s.*, not significant.

The activity of sensory fibers in the posterior lateral line nerve (PLLN) was recorded as multiple-unit spike discharge in the absence of hydrodynamic stimulation and during induced oscillatory translational motion to lateral line neuromasts on the tail with a sphere (Fig. 5A). The spontaneous firing without sensory stimulation (Fig. 5B_1_) was rather variable between different preparations with an average rate of ∼70 Hz (69.5±17.7 Hz; *n* = 11), depending on the number of simultaneously sampled units in a given recording. Generation of oscillatory translational water waves across the innervated skin area at 10 Hz (sphere displacement ±150 µm) evoked a rhythmic bursting that was strictly phase-locked to the cyclic stimulus (dashed lines in Fig. 5C_1_) with a peak firing rate of ∼140 Hz (143.2±24.2 Hz; *n* = 19). Bath application of 0.05% MS-222 (Fig. 5B_1_,C_1_) significantly reduced the spontaneous as well as the sensory stimulus-evoked firing after 5 min (*p*≤0.0001, *n* = 5) and caused a complete block of the multiple-unit nerve discharge after 15 min (Fig. 5B_2_,C_2_). The impact of MS-222 was more pronounced on the spontaneous compared to the sensory stimulus-coupled firing, however, the discharge was blocked completely under both conditions (Fig. 5D; n = 11). During wash-out, there was recovery of the spontaneous and evoked firing rate after 1 hour (Fig. 5B_1,2_,C_1,2_).

#### Vestibular nerve afferent activity

The activity of vestibular sensory afferents in the N.VIII was recorded as multiple-unit spike discharge in the absence of body motion and during sinusoidal horizontal-axis roll stimulation (Fig. 5E). The spontaneous (Fig. 5F_1_) multiple-unit N.VIII afferent firing rate of ∼15 Hz (14.7±6.5 Hz; *n* = 19) was modulated cyclically (Fig. 5G_1_) during sinusoidal turntable motion (0.5 Hz, ±10°), reaching an average peak firing rate of ∼70 Hz (71.4±17.2 Hz; *n* = 19). Since the N.VIII contains fibers from semicircular canal and otolith organs (Fig. 1B) with in part opposite directional sensitivities (i.e. anterior-posterior vertical canal, utricle), the multiple-unit peak response in the example shown in Fig. 5F,G was in phase with a horizontal-axis table rotation in the ipsi- as well as the contraversive direction (^#^ and ^##^, respectively, in Fig. 5G_1,2_). Application of 0.05% MS-222 (Fig. 5F,G) caused a gradual decrease of the resting rate and roll motion-evoked vestibular afferent firing to 20–30% of the control values after 5 min (*p*≤0.0001, *n* = 5) and a complete block of the spike activity after 15 min (Fig. 5F_1,2_,G_1–3_). This effect on the sensory nerve discharge was reversible, however the recovery was only partial after 1 hour wash-out reaching 20–30% of the control value (Fig. 5F,G). Determination of the dose-response curve of MS-222 (*n* = 19) for blocking spontaneous and roll motion-evoked vestibular afferent firing yielded a similar efficacy and dependency as observed for the corresponding lateral line activity (Fig. 5D).

## Discussion

Bath application of MS-222 and benzocaine, at variance with pancuronium, reversibly suppressed spontaneous and naturally evoked extraocular motor commands as well as responses in mechanosensory nerves in semi-intact *Xenopus* preparations in a dose-dependent manner, compatible with a specific block of voltage-dependent Na^+^-channels by both caine-derivates. The complete block of the discharge in sensory nerves by benzocaine and MS-222, along with the suppression of motoneuronal activity and muscle contractions indicates that these drugs have an analgesic, sedative and paralytic capacity and thus can be used as a single-drug anesthetic for surgical interventions in anamniotes. While the pharmaco-dynamics and efficacy of benzocaine and MS-222 were comparable, the use of an aqueous solution of the latter drug simplifies the anesthesia in fish and amphibians.

### Semi-intact in vitro preparations of Xenopus tadpoles for pharmacological studies of drug-related effects on neural processing and behavioral performance

The outcome of the present study was greatly facilitated by the employment of an isolated semi-intact preparation of *Xenopus* tadpoles that allowed *in vitro* neuropharmacology of a reactive sensory-motor behavior [14]. This preparation combines the advantage of *in vitro* drug testing such as the use of precise concentrations of substances and defined application onsets with the advantage of *in vivo* pharmacology with a specific behavioral readout. Classical pharmacological studies in contrast, are either performed *in vitro* on cell cultures (e.g. [23]), isolated parts of specific organs (e.g. [24]), or *in vivo* by behavioral pharmacology in the intact animal (e.g. [25]). Even though, each of the different methodological approaches has its advantages, all suffer from a variety of drawbacks, including difficulties in consolidating results from both approaches on concentration dependency, mechanistic principles or target sites of a specific drug. Neuroactive drugs usually influence the synaptic transmission or its modulation or impair the generation of action potentials. *In vitro* tests of such drugs, potentially interfering with single cell or network computations, typically employ cell cultures or slice preparations with limited residual interconnectivity between different cell groups [24]. While these studies allow evaluating dose-response curves or mechanistic principles, any inference on the behavioral impact of a particular drug remains difficult and requires additional *in vivo* studies. On the other side, a direct and unrestricted access to the central nervous system following systemic application of a neuroactive substance in intact animals is often prevented by the blood-brain barrier, thus impeding the assessment of behaviorally relevant effects of certain drugs.

While anamniote vertebrate species (fish, amphibians) are considered excellent model systems for drug discovery and testing [26], [25], isolated semi-intact preparations of larval or adult amphibians in particular combine many advantages of *in vivo* and *in vitro* approaches [20], [14]. The possibility to exploit specific behavioral paradigms, such as locomotion, respiration, vocalization or eye motion *in vitro* [27], [28], [18] and to record respective outputs as real or fictive motor behavior offers an unprecedented access to the underlying sensory-motor transformations for pharmacological impairment. This is of particular advantage for the evaluation of sites, mechanisms and concentration-dependency of general anesthetic drugs such as the caine-derivates in the current study. The unrestricted access to the central nervous system and the possibility to quantify a behavioral readout in an isolated preparation allowed precisely estimating the impact of the drugs on the spike activity of excitable cells, synaptic transmission and the generation of motor commands within a defined behavioral context. In addition, the intact sensory systems in *Xenopus in vitro* preparations faciliated a direct evaluation of the anesthetic drug effects on the encoding of spontaneous and evoked sensory signals at the first neuronal level. The reduction of spike amplitude and eventual block of the firing is compatible with the known mechanistic impact of caine derivates on voltage-gated Na^+^ channels [8], [29]. The block of Na^+^ conductances in all cells with excitable membranes (neurons and myocytes) also includes nociceptive sensory afferents, and thus, the employment of an isolated preparation of *Xenopus* tadpoles with intact sensory and motor systems for pharmacological studies provides indirect evidence for the analgesic potency of MS-222, supporting the ubiquitous use of this drug as single substance anesthetic for surgical interventions in fish and amphibians [6].

### Adequate anesthesia/sedation of anamniote vertebrates for surgical interventions

A number of different pharmacological substances, traditionally classified as anesthetics or hypnotic drugs, have been used previously to sedate anamniote vertebrate species for surgical interventions [1]. While anesthetic drugs such as MS-222 or benzocaine block action potentials in excitable cells, typical hypnotic drugs such as benzodiazepines or barbiturates have only indirect antagonizing effects on the spike discharge activity of neurons and neuronal networks. Both of the latter classes of drugs bind to respective sites of the GABA_A_ receptor, thereby potentiating the effect of GABA [30]. The consequence of a facilitation of the GABAergic inhibition is a sedative or hypnotic effect through a general reduction of neuronal excitability throughout the brain. A concomitant antagonizing influence of barbiturates on the glutamatergic AMPA receptor has been shown to further depress neuronal activity throughout the brain, thereby enhancing the sedative effect [31]. Despite the additional weak effect on nociception, barbiturates, however fail to offer a complete and reliable analgesia and therefore should not be used for surgical interventions in the absence of other analgesic compounds since anamniote vertebrates also possess pain receptors and pathways that are classically involved in the central nervous processing and perception of noxious stimuli [6], [32].

Apart from barbiturates and benzodiazepines, a number of anesthetic gases, such as metoxyflurane, halothane or isofluorane [33], [34] or injectable drugs including propofol, ketamine or tiletamine [35], [36], [37], [38] have previously been used for sedation in various anamniotes. Most of these sedatives however produce a variable depth and duration of anesthesia with an often unknown or unreliable analgesic effect [6]. While the detailed mode of action for most of these substances remains still unknown, all appear to exert their action through a more or less pronounced interference with various neurotransmitter or neuromodulator systems such as GABA, glycine, glutamate/NMDA or opiate receptors. A block or impairment of the neuronal transmission to various, partly unknown degrees however is inadequate for a reliable achievement and maintenance of a sufficiently deep and long anesthesia including analgesia and muscle relaxation. Thus, based on the broad mode of action that suppress the generation of action potentials in all nerve cells and muscle fibers, local anesthetics such as caine derivates (MS-222, benzocaine) are considerably more reliable and therefore more appropriate for the anesthesia in anamniote vertebrates for surgical interventions.

### MS-222 as potent single-substance anesthetics for anamniotes

In the current experiments on semi-intact *in vitro* preparations, MS-222 induced a complete block of eye motion, underlying extraocular motor commands but most importantly sensory nerve activity after 15 minutes of bath application. Accordingly, MS-222 exhibited classic sedative characteristics similar to ideal anesthetics, which are able to reversibly decrease body movements (paralytic), remove sensation (sedative/hypnotic) and relieve pain (analgesic), particularly during surgical interventions. Most local anesthetics have a similar mechanism of action and consist of a gradual reduction and finally complete block of action potentials [29]. The impairment of spike activity usually starts with small unmyelinated fibers and gradually blocks increasingly larger fibers, which in turn elicit a loss of sensation followed by a loss of motor function and CNS depression [1]. Since pain signals are transmitted in amphibians by small fibers, namely myelinated A-delta and unmyelinated C type fibers [39], there is a distinct advantage for MS-222 to block nociception in these animals at very early stages of drug exposure. Additionally, the duration of the MS-222 anesthesia is concentration/dose- and time-dependent. The concentration of MS-222 gradually increases in cells and tissue depending on the gradient. Since there is a higher extracellular concentration of MS-222, a large amount of the drug is absorbed, causing an increase in the drug effects. Similarly, the duration of exposure positively affects the extent of drug effect; the longer the exposure the longer the anesthetic effect lasts. Due to the accumulation of the drug in tissue and organs, there is a longer elimination time leading to an increased length of recovery time. However, when MS-222 is compared to another caine-derivate, benzocaine, its recovery time is considerably shorter. Since benzocaine is a lipophilic drug [40], it particularly sequesters in fatty tissue from where it is only released slowly and therefore its effects are not as readily reversible as those of MS-222. Thus, this makes MS-222 a very good choice of anesthetics, especially when experimentation requires a quick recovery of physiological function. On the other hand, MS-222 being highly water-soluble can present a problem for drug absorption, as it can be difficult for the drug to cross cell membranes given that they are not very permeable to ionized hydrophilic molecules. Fortunately, amphibians are equipped to readily absorb hydrophilic drugs in solution when submerged since they are capable of aqueous exchange across their gills and skin, at times through aquaporins [12], [41]. In fact, the water-solubility of MS-222 in contrast to benzocaine, which requires an organic solvent, is highly beneficial for a simple use in anamniote species. Nonetheless, both drugs have their distinct advantages as anesthetics in fish and amphibians and depending on the particular experimental condition and eco-physiological circumstance, the one or the other might be more appropriate, respectively. With respect to previous estimations of MS-222 for anesthesia, this drug has been proven in the current study to be an excellent single-drug anesthetic that includes appropriate analgesia and muscle relaxation for surgical interventions. MS-222 is thus well suited in particular for multiple anesthesia of individual animals or an employment in novel isolated anamniote experimental *in vitro* preparations [14], given the fact it completely blocks the spike discharge in muscle fibers, motoneurons and sensory neurons.
